# A Study of Erosion–Corrosion Behaviour of Friction Stir-Processed Chromium-Reinforced NiAl Bronze Composite

**DOI:** 10.3390/ma15155401

**Published:** 2022-08-05

**Authors:** Varun Dutta, Lalit Thakur, Balbir Singh, Hitesh Vasudev

**Affiliations:** 1 School of Mechanical Engineering, Shri Mata Vaishno Devi University, Katra 182320, India; 2 School of Energy Management, Shri Mata Vaishno Devi University, Katra 182320, India; 3 Mechanical Engineering Department, National Institute of Technology Kurukshetra, Kurukshetra 136119, India; 4 School of Mechanical Engineering, Lovely Professional University, Phagwara 144411, India

**Keywords:** friction-stir processing, weight loss, corrosion resistance, microstructure

## Abstract

Corrosion is frequently viewed as a catastrophic and unavoidable disaster in marine applications. Every year, a huge cost is incurred on the maintenance and repair of corrosion-affected equipment and machinery. In the marine environment, as-cast nickel–aluminium bronze (NAB) is susceptible to selective phase corrosion. To solve this problem, chromium-reinforced nickel–aluminium bronze was fabricated using the friction stir process (FSP) with improved microstructures and surface properties. A slurry erosion–corrosion test on as-cast and FSPed composites demonstrated that the developed surfaced composite has lower erosion and corrosion rates than the as-cast NAB alloy. The erosion–corrosion rate increased with a decrease in the impact angle from 90° to 30° for both as-cast NAB and prepared composites, exhibiting a shear mode of erosion. The specimens at impact angle 30° experienced more pitting action and higher mass loss compared with those at impact angle 90°. Due to increases in the mechanical properties, the FS-processed composite showed higher erosion resistance than the as-cast NAB alloy. Furthermore, corrosion behaviour was also studied via the static immersion corrosion test and electrochemical measurements under 3.5 wt.% NaCl solution. In a static immersion corrosion test, the FSPed composite outperformed the as-cast NAB composite by a wide margin. The FSPed composite also demonstrated a reduced electrochemical corrosion rate, as revealed by the polarization curve and electrochemical impedance spectroscopic (EIS) data. This reduced rate is attributed to the formation of a Cr oxide film over its surface in the corrosive environment.

## 1. Introduction

Erosion is the process of removing the surface layer of materials that are directly exposed to gas and liquid. Furthermore, eroded particles impinge on these materials and lead to the removal and decaying of the material inwardly [1]. Erosion is a complicated process since it involves numerous factors, each of which has a significant impact when taken together [2].

The corrosion of a material is one of the most common degradation issues limiting the materials’ efficiency and service life. Corrosion leads to the decaying of a material on account of its chemical reaction with the surroundings. Mainly, corrosion occurs in metals when their surface comes into direct contact with liquid or gas, and it further deteriorates in environments with warm acids, salts, and high temperatures [3].

A combined erosion–corrosion mechanism accelerates material removal on a surface, especially in marine environments. It poses the most severe problem to the marine industry, as ship propellers, rudders, etc., are exposed to seawater, degrading their efficiency and longevity with time. These failures, downtime and material replacement, heavily increase costs on the maintenance/operational budget of the marine industry. Erosion–corrosion causes significant damages/losses to parts beyond economical repair [4,5,6].

Many researchers have explored this vulnerable erosion–corrosion phenomenon in marine applications. Various studies were performed on erosion–corrosion using the slurry impact with different erodants of different shapes and sizes. The effects of impact velocity, slurry impingement angle, and different types of erodants on marine materials were studied and reported in the literature [7,8,9,10]. Moreover, various researchers have coated base metals to enhance their erosion-resistance properties [11,12,13,14,15].

Nickel–aluminium bronze (NAB) alloy is frequently utilized in the marine industry due to its high cavitation erosion and corrosion resistance in seawater [16]. It is used to make propellers, pipelines, valves, and pumps. However, NAB degrades over time in marine environments, and the surface layers of the best corrosion-resistant alloys can be used to prevent direct contact of NAB with liquids and gases [17]. The friction stir process (FSP) is a potential technique for enhancing a material’s erosion and corrosion resistance by improving the microstructure and by reducing the porosity problem [18]. In the FSP, a non-consumable tool under an axial force is rotated and moved over the surface of the clamped workpiece. Due to frictional heat between the tool and the workpiece, significant grain refinement and plastic deformation are achieved. Various researchers have also shown that the FSP has refined and homogenized the microstructure of NAB alloys [19,20,21]. Song et al. [22] performed a multi-pass FSP on NAB and reported improved erosion and corrosion resistance. It was also reported that the FSP removed the casting defects of the NAB alloy [23,24,25].

A NAB surface composite can be fabricated via friction stir processing to enhance the corrosion and erosion resistance of an as-cast NAB alloy. The microstructure and corrosion characteristics of FSPed NiAl bronze were investigated by Lv et al. [25]. The microstructure of a NAB alloy is significantly affected by FSP processing parameters. It was claimed that, by lowering the tool’s rotation with constant traverse speed, the corrosion properties were improved. FSP was used by Abbasi and Keshavarz [26] to fabricate a NAB/Al_2_O_3_ surface nanocomposite. Nanosized Al_2_O_3_ reinforcement was used to study the microstructure, hardness, and corrosion resistance of an alloy surface. The corrosion resistance of the alloy surface improved significantly. Zhong et al. [27] investigated the impact of heat treatment on the erosion–corrosion behaviour of NAB in a chloride solution. Heat treatment had no discernible effects on the electrochemical polarization behaviour of the NAB alloy, while it was demonstrated that adding more hard phases, such as β’, can increase the NAB alloy’s resistance to erosion–corrosion.

Various researchers have reported that properties such as strength, wear resistance, and corrosion and erosion resistance have improved with the addition of chromium in these metals and alloys. A Cr-rich oxide film is formed on the surface of these metals and alloys, thus preventing direct contact of the corrosive medium with the base material [28,29,30,31,32,33,34]. Qin et al. [35] attempted chromium ion implantation on the surface of NAB to modify its surface microstructure. Other researchers studied its electrochemical behaviour and performed a salt spray test on its surface. It was reported that corrosion resistance was improved on account of the development of chromium oxides and hydroxides on the surface of NAB. Many studies have found that adding Cr to nickel, stainless steel, and aluminium alloys improved their corrosion resistance [36,37,38,39].

Previous studies were mostly conducted to assess the mechanical, tribological, erosion–corrosion, and microstructural properties of NAB. FSPed NAB was reported with enhanced properties. Chromium reinforcement in a NAB material using the FSP may improve its mechanical and erosion–corrosion behaviours. In the present study, chromium-reinforced nickel aluminium bronze is fabricated via the friction stir process. As-cast NAB and a processed composite were subjected to slurry erosion–corrosion tests, immersion corrosion tests, and electrochemical tests to study their corrosion and erosion behaviours for marine applications.

## 2. Experimentation

### 2.1. Materials and Friction Stir Processing

**Figure 1:**
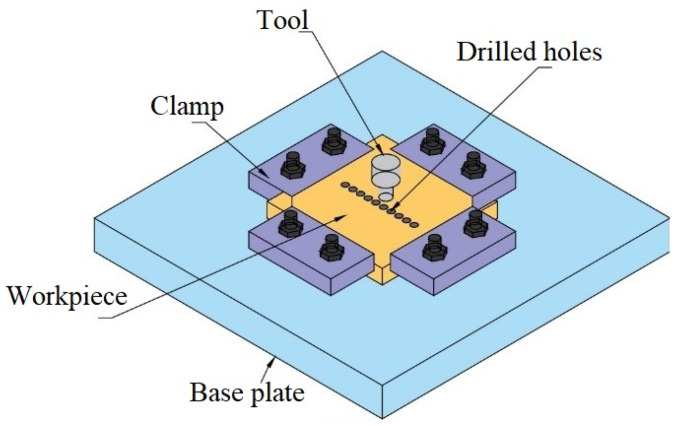
Fabricated setup for the FSP.

The multi-pass FSP was performed on NAB using chromium (Cr) as reinforcement. The chemical composition of the NAB alloy provided by M/s Govind Metals Co., Gujarat, India, was 79.8 wt.% Cu, 9.9 wt.% Al, 3.5 wt.% Fe, 1.2 wt.% Mn, and 5.6 wt.% Ni. Chromium powder with a particle size range of 10–50 μm and 99% purity was supplied by M/s Om Enterprise, Gujarat, India. Several holes 2 mm in diameter, 6 mm in depth, and 2 mm apart were made on the surface of the NAB plate and then filled with the Cr powder. This configuration incorporated a 12.6% volumetric concentration of Cr reinforcement in the NAB base material. A special fixture (Figure 1) was fabricated to hold the plate firmly on a vertical milling machine (Batliboi BFV-5 Vertical Milling Machine, Katra, India) for the FSP. A tool made of Inconel 718 (M/s Bharat Aerospace Metals, Mumbai, India) material with a taper-shaped pin (tip diameter of 4 mm, base diameter of 6 mm, and length of 5 mm) was used to perform the four-pass FSP. The FSP parameters used were a tool rotation of 1000 r.p.m., a tool feed rate of 28 mm/min, and a tilt angle of 0°. The specimens were sectioned from the as-cast NAB and the FSPed composite to conduct a micro-hardness test using a Digital Micro Hardness Tester with a load of 300 gm and a dwell time of 20 s. Tensile specimens were sectioned from the stir zone using wire cut EDM in accordance with the E8 ASTM standard and tested on a Universal testing machine (Dak Inc., Model 7200, Ahmedabad, India).

### 2.2. Surface Characterization

The specimens for metallographic examination were sectioned from the processed zone. Specimens were mounted in epoxy resin and polished with SiC papers (320 grit to 2000 grit) and alumina powder. Etching was performed with a solution of 2 mL of HCl, 5 g of FeCl_3_, and 95 mL of ethanol, followed by rinsing with acetone and distilled water. The microstructures of the as-cast NAB, Cr powder, and FS-processed composite were studied with a field emission scanning electron microscope (JOEL JSM 7900F, Jammu, India) fitted with energy dispersive spectroscopy (EDS). Three EDS measurements at different spots were taken for each sample to ensure the validity of the compositional results. The elemental composition, reported as a minimum of two results, are shown in Section 3. The image analysis was performed to measure the particle size using the software (Image J). The surface morphology of the tested specimens was also examined to study the erosion and corrosion mechanisms. To identify the phases contained in the as-cast NAB and fabricated composite, an XRD analysis was performed using a Bruker D8 advance diffractometer. Cu-Kα radiation from a 1.54 Å source was used for the XRD examination, with a scan range of 10°–100° and a scan speed of 2° per min.

### 2.3. Slurry Erosion–Corrosion Testing

**Figure 2:**
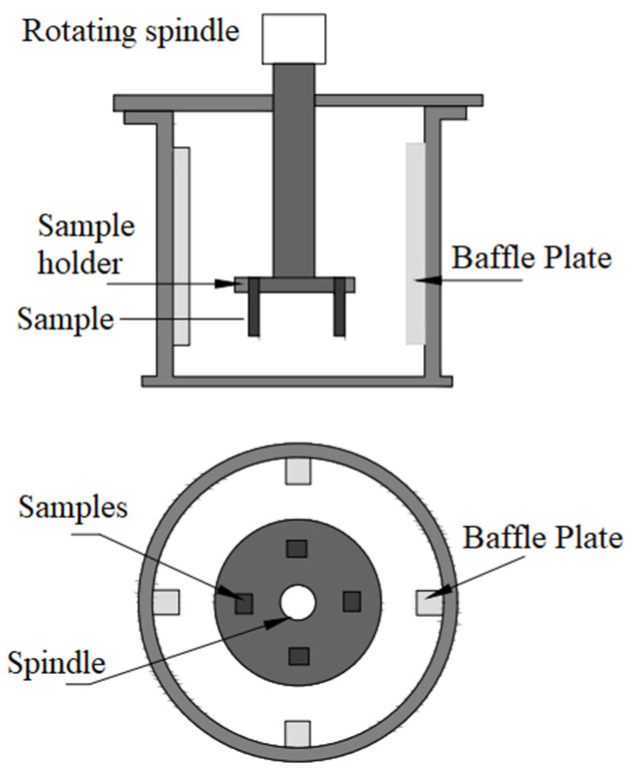
Slurry erosion test rig.

Test samples with dimensions of 10 mm by 10 mm were cut from the processed zone to conduct slurry erosion–corrosion testing with the help of the wire-cut EDM process. The specimens were polished with SiC abrasive papers (grit size from 320 to 1500) and 1 µm of alumina paste on a double-disc polishing machine and rinsed with acetone and water. The specimens were initially weighed and placed in the sample holder of the specially designed test setup shown in Figure 2. Various parameters such as impact angle, impact velocity, and mass flux (m) could be varied in this setup. The slurry mixture consisted of distilled water, silica sand (size 300–500 µm), and NaCl in the ratio of 10:3:2. The tests were conducted at different impact angles by changing the orientation of the specimens in the holder for 5 h. A testing period of 5 h was considered a suitable amount of time allowed for the specimens to reach a steady state.

After testing, the samples were cleaned thoroughly with deionized water and degreased with acetone to remove any embedded erodent and corrosion substance. Then, the samples were dried in air and weighed on a weighing machine with 0.001 gm least count. Measurements were taken three times, and the average weight loss was calculated. The following equation was used to determine the corrosion rate [40]:

(1) $$\mathrm{Corrosion}\;\mathrm{rate}=\frac{k\times △w}{a\times t\times \rho }\;\mathrm{mm}\;\mathrm{per}\;\mathrm{year}\;(\mathrm{mm}\;y^{-1})$$

where k is a constant (87,600), Δw is the mass loss (grams), a is the exposed area (cm^2^), t is the time of exposure (h), and *ρ* is the density (g cm^−3^).

### 2.4. Static Immersion Corrosion

Specimens of length 6 mm, width 6 mm, and thickness 7 mm were machined from the stir zone for immersion. These were polished with (1000 grit) SiC abrasive paper, degreased with acetone, dried with a blower, and weighed to 0.001 gm precision. The specimens were suspended and immersed in a tank containing 10 L of 3.5% NaCl solution for 1, 2, 4, 8, and 16 days, according to the ASTM G31-72 standard [41]. To remove the corrosion products, the corroded samples were rinsed with water and submerged in a solution with 500 mL of HCl (density, 1.19) and 1000 mL of H_2_O for 2 min. Again, the specimens were thoroughly cleaned with a brush and rinsed with distilled water. Finally, the specimens were immersed in ethanol and blow-dried before being weighed. The weight reduction was calculated by averaging three findings for each condition. The morphology of the corroded surfaces was studied using an optical microscope (OM), Kurukshetra, India and scanning electron microscope (SEM). The corrosion rate based on weight loss was calculated using the following formula:

$$\mathrm{CR}=\frac{\Delta m}{S\times t}$$

where CR is the corrosion rate in mg.cm^−2^. day^−1^, Δm is the mass loss, S is the area of the exposed surface of the sample in cm^2^, and t is the time duration in days.

### 2.5. Electrochemical Testing

Specimens of sizes 20 mm × 20 mm × 8 mm were sectioned for corrosion testing of the as-cast NAB and processed composite. The specimens were polished with SiC abrasive paper (from grit size 320 to 2000), washed with distilled water, and dried in air. The specimens were masked to achieve an active area of 6 mm^2^ for the testing. 

**Figure 3:**
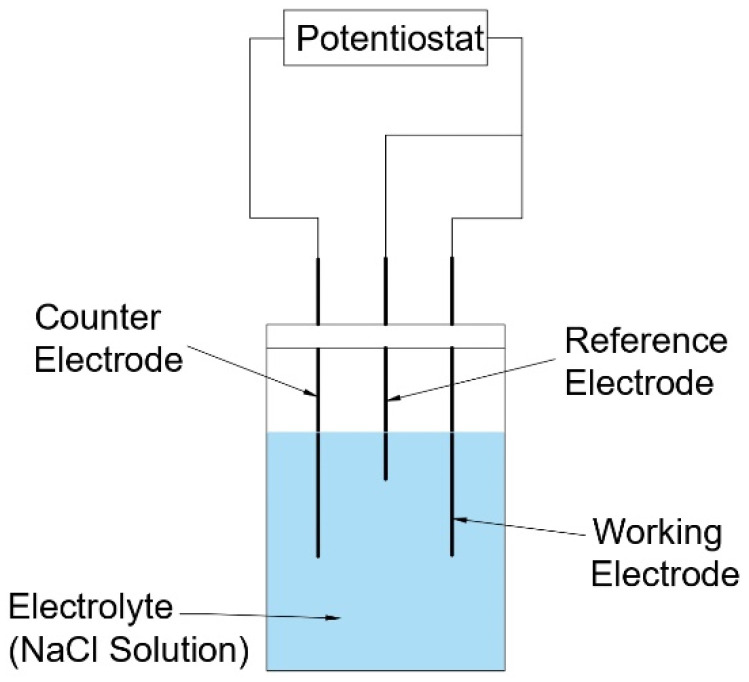
Schematic diagram of the three-electrode setup for electrochemical testing.

The electrochemical testing was conducted in a 3.5 wt.% NaCl solution. The electrochemical behaviours of the as-cast NAB and the prepared composite were determined as per ASTM standards (G5-94 and G-61-86) at room temperature. A potentiostat (make biologic, model SP 200, Kurukshetra, India) consisting of a three-electrode cell setup was used to perform the test. The reference electrode used was a saturated calomel electrode (SCE), a platinum mesh wire served as a counter electrode, and the working electrode was the specimen to be studied. A schematic diagram of the three-electrode setup is shown in Figure 3. Before performing the actual test, the specimens were held in the test solution for 500 s at resting potential to achieve a stable open circuit potential. For evaluating the electrochemical corrosion behaviour, potentiodynamic polarization curves were analysed using EC-lab software. The anodic and cathodic Tafel slopes were obtained by measuring the polarization curve at a rate of 0.5 mV/s. Electrochemical impedance spectroscopy (EIS) was measured with a 5 mV AC current signal in the frequency range from 10 MHz to 100 kHz. The test results of an average of three specimens were taken to ensure accuracy. Polarization resistance (R_p_) was determined by performing a linear potentiodynamic sweep with a range of ±5 m V_OCP_ at a rate of 0.33 mV/s.

## 3. Results and Discussion

### 3.1. Microstructural Characterization

**Figure 4:**
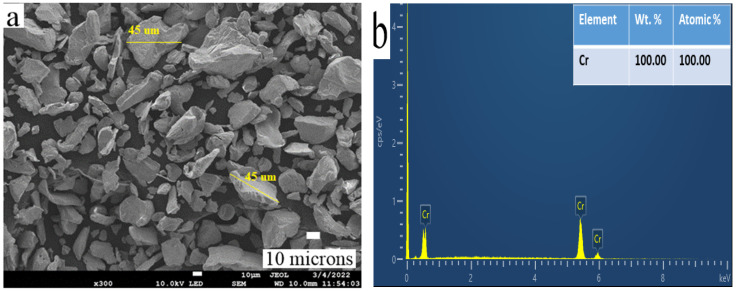
(**a**) FE-SEM image of Cr powder and (**b**) EDS image of Cr powder.

SEM was performed on the chromium reinforcement powder, showing an irregular shape, as shown in Figure 4a. The EDS analysis (Figure 4b) confirmed the presence of chromium particles. An image analysis confirmed the particle size of Cr in the range of 10–50 μm, as certified by the supplier.

**Figure 5:**
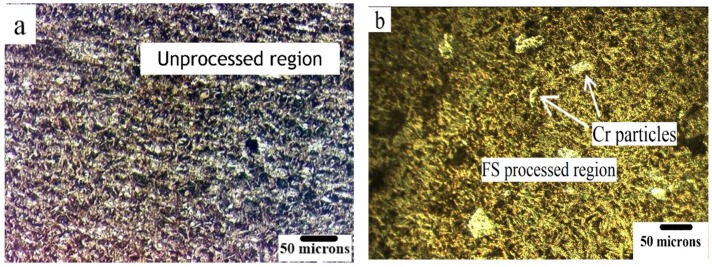
Optical micrographs of (**a**) the as-cast NAB and (**b**) FS-processed stir zone showing the distribution of particles.

Optical microscopy images of the as-cast NAB and the FSPed composite are shown in Figure 5a,b The grain size was measured from the image analysis, and it was revealed that the grain size was reduced from 20 μm to 6.3 μm, exhibiting significant improvement after the FSP.

**Figure 6:**
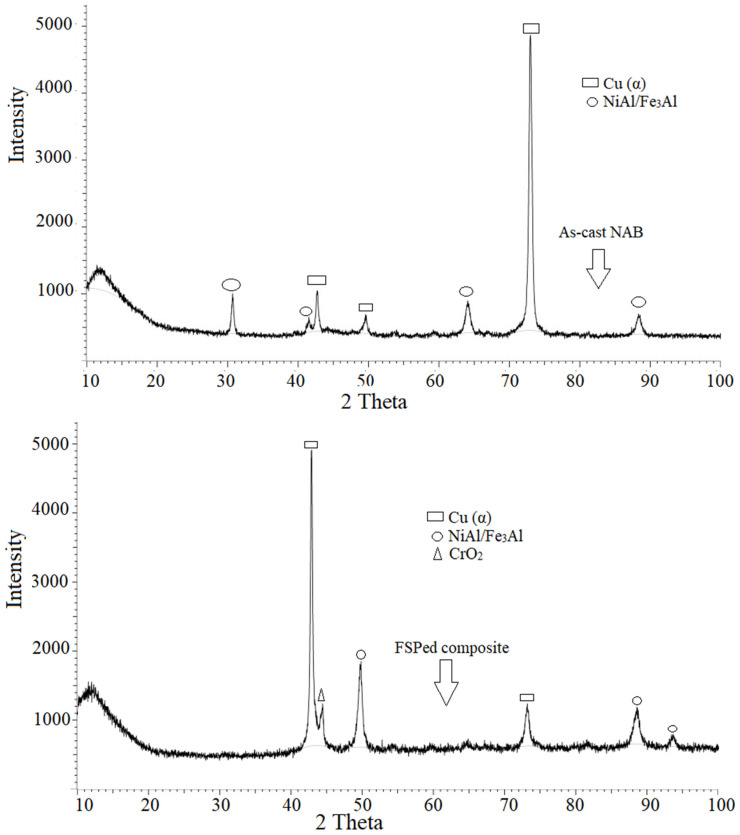
XRD graph of the as-cast and FSPed composites.

Figure 6 displays the X-ray patterns of the as-cast NAB and the FSPed composites to show their phase structures. The majority of the peaks correspond to the NAB alloy’s FCC α phase. Due to residual strain in the processed area during the FSP, the peak shifted and chromium oxide formation was noticed. The existence of a CrO_2_ phase validated its presence and confirmed the formation of a protective layer on the surface of the developed composite. Some peaks in the NiAl/Fe_3_Al phase disappeared in the FSPed composite when compared with the as-cast NAB. However, new peaks have also emerged on account of disorder in the atomic arrangement caused by frictional heat and severe plastic deformation during the FSP.

Both the as-cast NAB and the FSPed composite reported no undesired phases. The normalized intensity ratio (NIR) [42] of the phases was calculated using the following relation for a semi-quantitative analysis of the various phases present in the as-cast and FSPed composites:

$${\;\mathrm{NIR}}_{1}=\frac{I_{1}-I_{\mathrm{back}}}{I_{1}+{\;I}_{2}+I_{3}-{\;I}_{\mathrm{back}}}$$

where I_1_, I_2_, and I_3_ represent the intensities of first, second, and third phases, respectively, and I_back_ indicates the background intensity. The above equation was further employed for calculating the NIR value for all phases. The major peak corresponding to that particular phase was taken from the XRD spectrum for calculating the NIR of various phases. In the NIR method, only the relative amount of various phases present can be calculated because the NIR method does not provide the precise amount of phases present in the as-cast NAB and the FSPed composite. The approximate values of the phases are presented in Table 1. The phase intensity of the phases for Cu, NiAl/Fe_3_Al, and CrO_2_ are 67.63%, 21.03%, and 11.33%, respectively. On the other hand, the as-cast NAB showed phase intensities in Cu and NiAl/Fe_3_Al of 95.15% and 4.85%, respectively.

**Figure 7:**
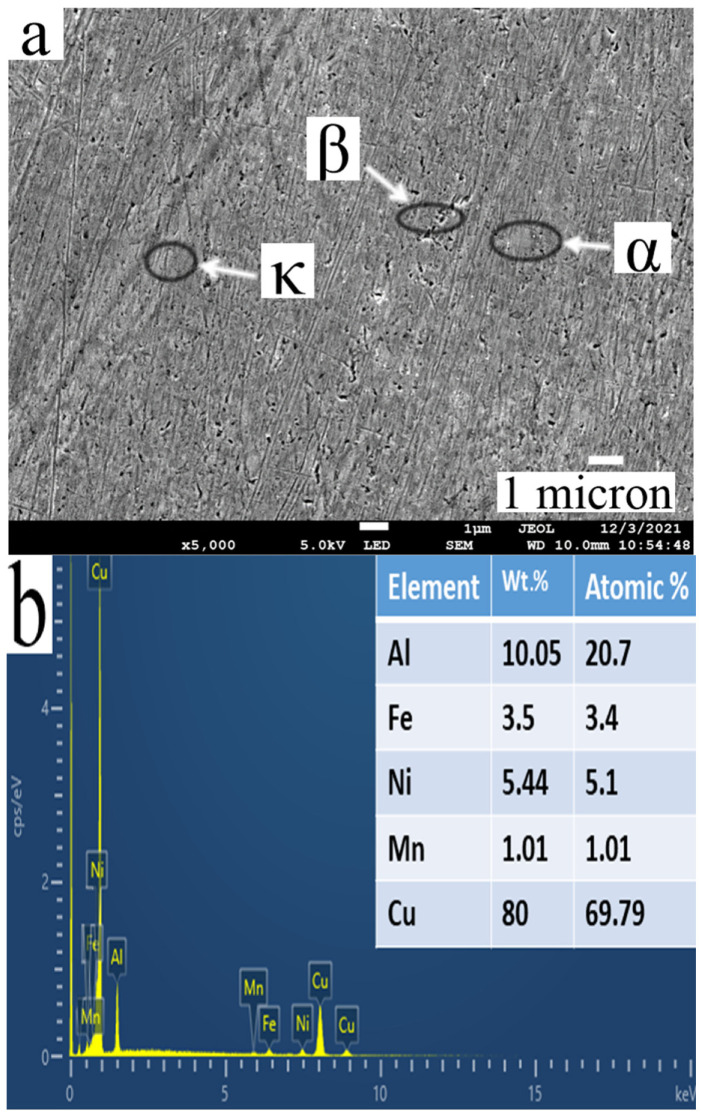
(**a**) FE-SEM image of as-cast NAB and (**b**) EDS image of as-cast NAB.

The FE-SEM images of the as-cast NAB show α, β, and κ phases performed at 5000× magnification, as shown in Figure 7. The primary coarse α phase is rich in Cu, and the κ phases comprise κIII (lamellar shape), which is rich in NiAl and κII (globular shape) and κIV (globular shape), which are rich in Fe_3_Al. However, no iron-rich κI phase (rosette shape) is observed as it occurs only when the iron content in the alloy is more than 5% (Figure 7).

**Figure 8:**
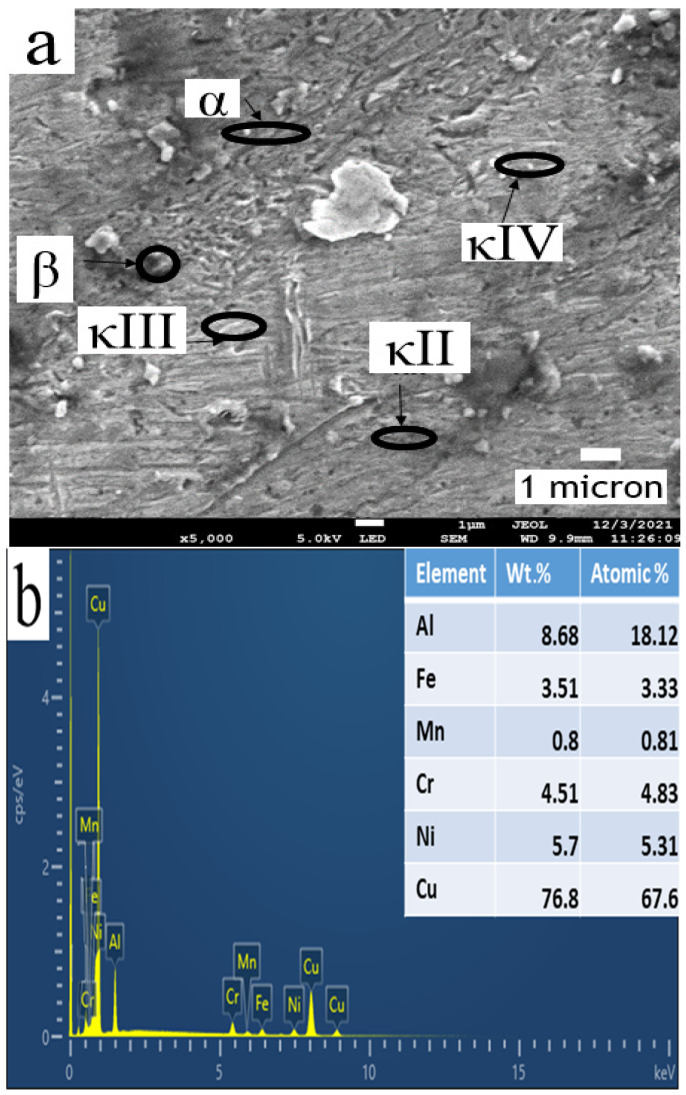
(**a**) FE-SEM image of the FSPed NAB–Cr composite and (**b**) EDS image of the FSPed NAB–Cr composite.

**Figure 9:**
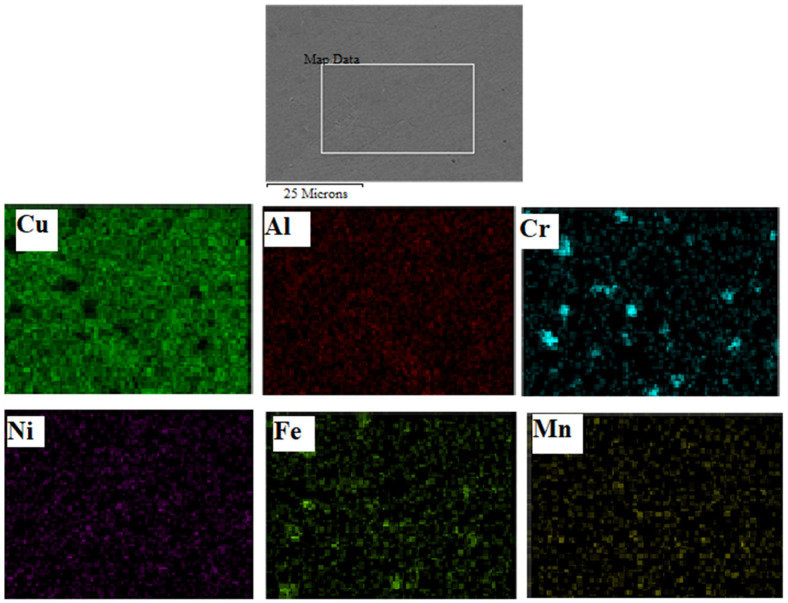
EDS mapping of all of the elements in the FSPed composite.

During the FS process, the Cu-rich α and the eutectoid regions in the as-cast NAB undergo gradients due to high temperatures in severely affected areas near the stir zone. Due to the FS process, the α and β phases are broken down into finer the globular and elongated phases [34,43]. Additionally, due to severe plastic deformation in the FS process, the κ phases are homogenized (Figure 8a). The energy dispersive spectra (EDS) confirm the fabrication of a NAB composite with chromium addition, as depicted in Figure 8b. EDS elemental mapping was performed on the Cr-reinforced NAB composite at the specific area shown in Figure 9. Cu, Al, Ni, Fe, Cr, and Mn are the primary elements that are seen in the mapping. Additionally, the reinforcement is uniformly dispersed in the NAB matrix of the processed zone.

### 3.2. Slurry Erosion–Corrosion Test

**Figure 10:**
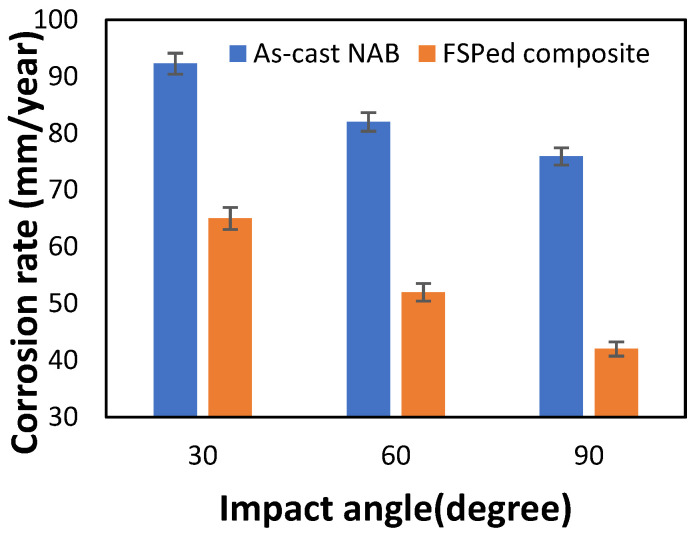
Corrosion rate vs. impact angle.

The weight losses obtained after the slurry erosion test were measured for the as-cast and FSPed composites. The tests were conducted for 30°, 60°, and 90° impact angles. It was observed that the weight loss for impact angle 30° was more in comparison with impact angle 90°, as mentioned in Table 2. The erosion and corrosion rates of the as-cast NAB and the processed composite were calculated using Equation (1). The computed values are represented by a histogram, as shown in Figure 10. It was observed that as-cast NAB exhibited a high erosion–corrosion rate when compared with the FSPed composite.

The coarse microstructure of the as-cast NAB led to the segregation of constituents, thereby providing more passage for the corrosive solution to attack the various phases. This resulted in a decrease in corrosion resistance. In contrast, the FSP facilitates the breakdown of coarse phases, leading to the homogenization of the constituents. This minimizes passage for the corrosive solution, thereby enhancing the corrosion resistance, as reported by various authors [1,19,20,21]. This may be attributed to the fact that the FSPed composite has a fine and homogenized grain structure compared with the as-cast NAB. The FSPed surface has a significant level of residual stresses and strains, which leads to a uniform corrosion rate [19,25]. Furthermore, the increased corrosion resistance is due to the formation of a more protective layer containing CrO_2_. Additionally, the microhardness results showed that as-cast NAB has a hardness value of 286 HV, which increased to 385 HV, as obtained from the chromium-reinforced FSPed composite. The treated composite showed an ultimate tensile strength of 701 MPa, whereas the as-cast NAB exhibited an ultimate tensile strength of 620 MPa. The percentage elongations of the as-cast NAB and the FSPed treated composite are 12% and 16.4%, respectively. The improved hardness value, tensile strength, and percentage elongation of the FS-processed composite led to a reduction in erosive wear [44,45].

As seen in Figure 10, the corrosion rate increased with a decrease in impact angle from 90° to 30°. Since the erosion was low at a high impact angle (90°) and significantly high at a low impact angle (30°), the NAB material exhibited a shear mode of erosion. At a 90° impact angle, normal stress was predominant, and the surface film was damaged but remained on the surface, providing protection from the corrosive environment. At a low impact angle, shear stress dominates, causing the material to shear and the oxide film to be removed from the surface [46,47].

**Figure 11:**
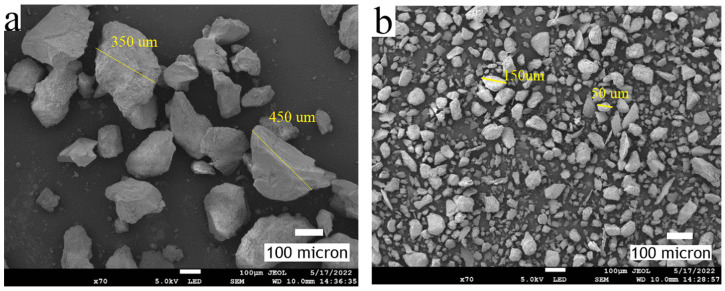
SEM micrographs of silica sand (**a**) before the test and (**b**) after the test.

Silica sand was analysed using a scanning electron microscope (SEM) at 70× magnification before and after the slurry erosion–corrosion test, and the micrographs are shown in Figure 11a,b. It can be observed that, before the test, the average particle size of the silica sand was large and that the particles were sharp in comparison with the particles obtained after performing the test. The average particle size before the slurry erosion–corrosion test was 300–500 μm, whereas, after completing the test, it was reduced to 50–300 μm.

**Figure 12:**
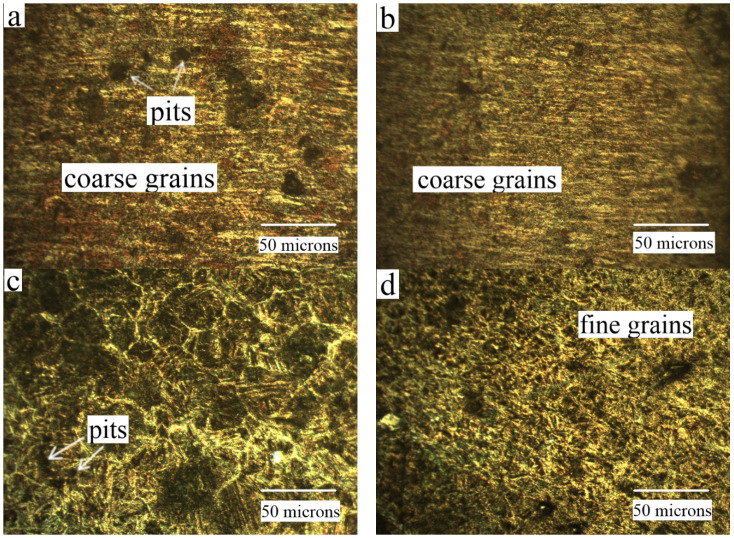
Optical micrograph of slurry erosion–corrosion for (**a**) as-cast NAB at 30°, (**b**) as-cast NAB at 90°, (**c**) FSPed composite at 30°, and (**d**) FSPed composite at 90°.

Optical images of the specimen after slurry erosion–corrosion of the NAB alloy and the composite at impact angles 30° and 90° are shown in Figure 12. The specimen at impact angle 30° shows more pitting action than the specimen at 90° impact angle. This was also revealed in the case of a higher mass loss at 30° impact angle and a lower mass loss at impact angle 90°. It can be seen that the microstructure of the as-cast substrate has a coarse grain structure in comparison with the FSPed composite, which contains a fine homogeneous structure [48,49,50].

Due to the coarse structure in the as-cast NAB alloy, there is segregation of elements mainly in the grain boundaries, providing the path for the corrosive solution to attack. However, the coarse microstructure is broken down due to the effect of the stirring action of the tool, resulting in a fine and homogeneous structure. Thus, the corrosion path is eliminated and thereby decreases the corrosion attack on the substrate. The exceptional performance of a Cr-reinforced NAB composite can be attributed to the formation of a protective film composed of copper and chromium oxides.

**Figure 13:**
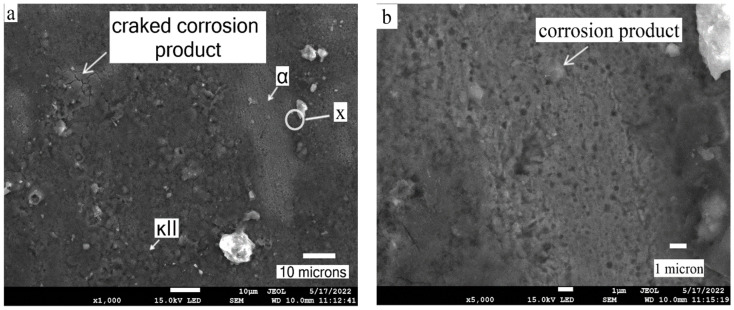

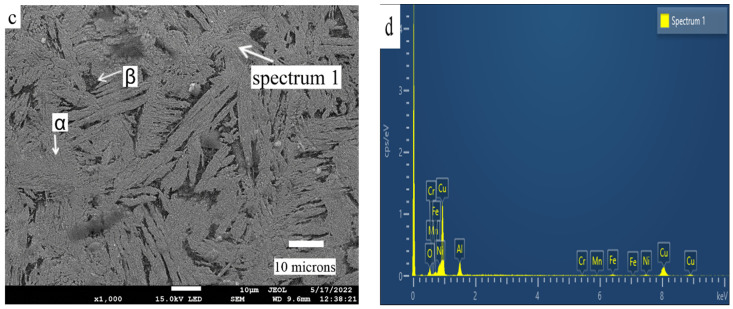
Scanning electron microscope morphology of slurry erosion–corrosion for (**a**) as-cast NAB at 30° (1000×), (**b**) magnified image at X (5000×), and for (**c**) FSPed composite at 30° (1000×), (**d**) EDS image of spectrum 1.

SEM images of the as-cast NAB and the processed composite taken after the slurry erosion–corrosion test at 30° impact angle are shown in Figure 13a–c. In the case of as-cast NAB, the passive film degraded or was damaged by the high impact kinetic energy of the slurry particles, leading to an increase in erosion–corrosion. The images clearly showed the effect of corrosion in both the as-cast NAB and the processed composite. Due to grain refinement of the FSPed composite, its structure was well organized, which reduced the effect of erosion–corrosion compared to the as-cast NAB. The FSPed surface of the composite contains high residual stress and strain, promoting a uniform corrosion rate. The presence of Cr in the FS surface reduces the erosion–corrosion rate. Moreover, an increase in hardness in FS-processed composite also leads to a decrease in erosion–corrosion. The EDS image revealed the presence of all of the elements in the processed composite after corrosion, as shown in Figure 13d.

### 3.3. Static Immersion Corrosion

**Figure 14:**
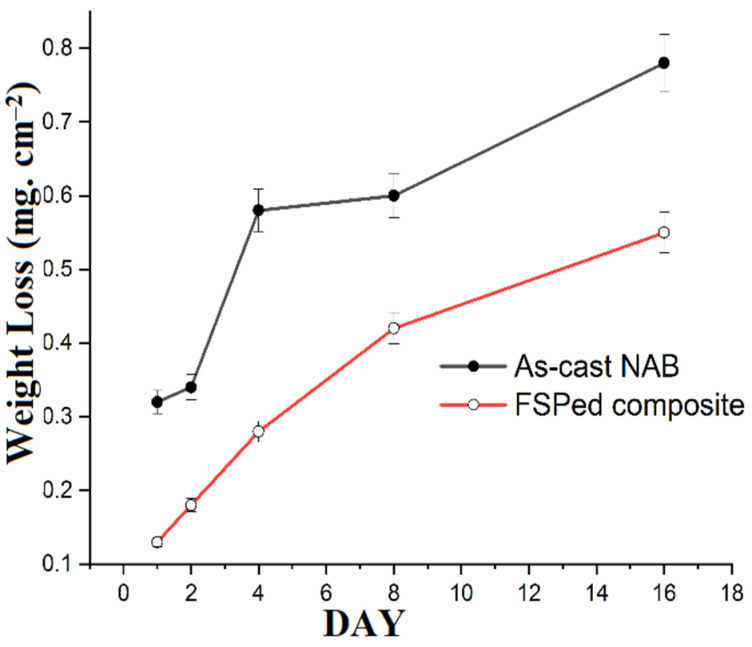
Weight loss in as-cast NAB and FSPed composite specimens after conducting static immersion tests in 3.5% NaCl solution.

**Figure 15:**
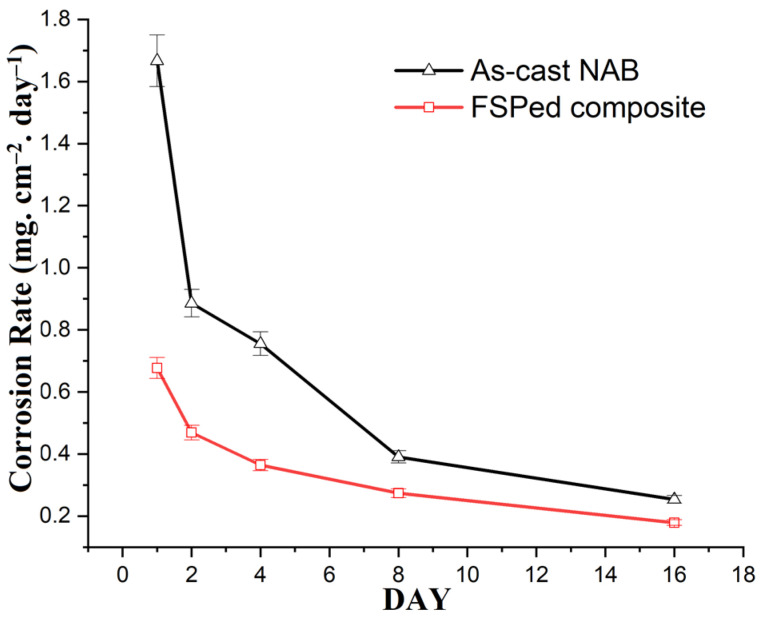
Corrosion rate of as-cast NAB and FSPed composite specimens after conducting static immersion tests in 3.5% NaCl solution.

Figure 14and Figure 15 illustrate the weight loss and corrosion rate of the as-cast NAB and FSPed composites, respectively. It is observed that the weight loss and corrosion rate of as-cast NAB were higher than those of the FSPed composite in the static immersion test. The corrosion rate of the specimens decreased from Day 1 to Day 16. Moreover, the weight loss and corrosion rate appeared to be more steady from Day 8 onwards for all of the specimens. This may be attributed to the formation of protective surface films on the specimens. The copper alloys in the chloride medium formed protective films while coming in contact with the chloride medium [51,52,53]. Cuprous dichloride is believed to be formed during the process due to the anodic dissolution of copper in the NAB corrosion process, as reported by some researchers [41,42].

Moreover, owing to the presence of Al in the NAB alloy, oxide and hydroxide of the hydrated aluminium layer were formed in the chloride solution. These protective surface layers containing copper and aluminium enhanced the corrosion resistance [54,55]. These protective films acted as barriers and protected the base material from damage due to a corrosive medium. The enhanced resistance in the FSPed composite is attributed to the creation of a more protective surface layer containing chromium oxides and hydroxides. The decrease in corrosion of the FSPed composite was on account of the following factors.

First, the as-cast NAB alloy’s coarse microstructure has a lower corrosion resistance than the homogenized finer microstructure of the FS-processed composite. The size, structure, and distribution of coarse grains in the as-cast NAB affect the corrosive activity significantly. Due to the coarse microstructure, segregation of the constituents takes place in the grain boundaries, thereby providing a path to the corrosive solution [56]. Second, the FS process reduces the size and improves the distribution of fine grains, thereby facilitating the homogenization of the constituents. This significantly reduces the path for the corrosive solution, hence decreasing the corrosion rate. Third, the casting process gives rise to porosities in the as-cast NAB, thus increasing the path between the substrate and the corrosive medium, leading to a surge in corrosion. The FS process eradicates porosity defects, thus decreasing corrosive effects significantly. Furthermore, with an increase in the immersion duration, the corrosion rate of the as-cast and FSPed composites decreased on account of the formation of protective surface films [52,57,58]. This layer acts as a barrier and hinders the action of the corrosive medium, thus increasing the corrosion resistance.

**Figure 16:**
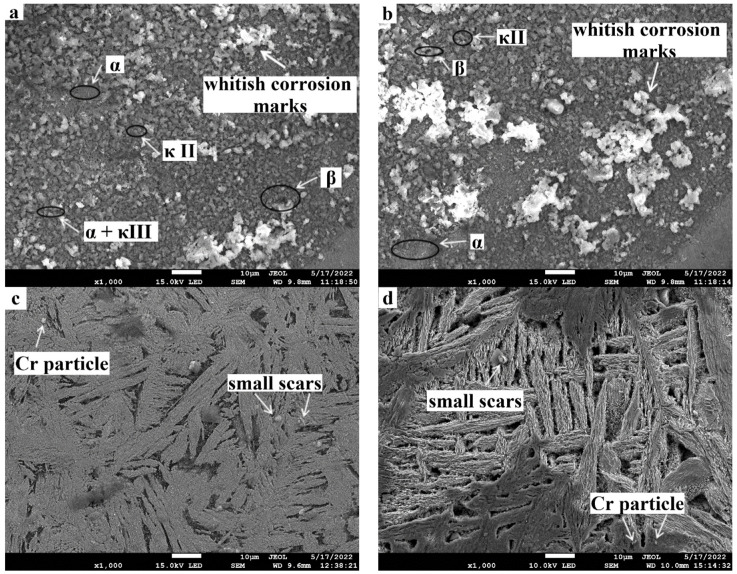
SEM micrographs of (**a**) as-cast NAB specimen surfaces after exposure for 1 day, (**b**) as-cast NAB specimen surfaces after exposure for 16 days, (**c**) FSPed composite after exposure for 1 day, and (**d**) FSPed composite after exposure for 16 days in 3.5% NaCl solution.

Figure 16a–d show the surface morphology of post-immersion corrosion samples at magnification of 1000×. After Day 1, the as-cast NAB specimen displayed noticeable corrosion, having whitish corrosion marks of 10 µm in size scattered on the surface film (Figure 16a). Furthermore, the presence of some pores was seen on the surface. The FSPed composite specimen had less corrosion than the as-cast specimen corroborated by a few corrosion scars visible on the upper surface.

After Day 16, corrosion was predominantly on both the as-cast and FSP specimens. For the as-cast NAB specimen, both the extent and the area of the scars were apparently enlarged, with some bigger ones being 50 µm in size, as shown in Figure 16d. The FSP specimen contained small scars compared with the as-cast NAB. The surface film of the as-cast NAB was rough, and regions containing κ phases were corroded more than those with the α phase. The κ phases have a coarse structure and show a non-uniform distribution, thereby promoting corrosion. As observed in Figure 16b, the shrinkage cavities were seen in the as-cast NAB, which are not visible in the FS-processed composite. The presence of the shrinkage cavities enhances the corroded surface in the as-cast NAB. The FS process alters the morphology of κ phases and uniformly segregates grain dispersion, thus lowering corrosion rates [59,60]. When NAB alloy comes into contact with a corrosive solution, copper oxides form, providing corrosion protection. Additionally, the presence of chromium results in the formation of oxides and hydroxides on the surface layers, thus enhancing corrosion resistance.

### 3.4. Electrochemical Corrosion Results

**Figure 17:**
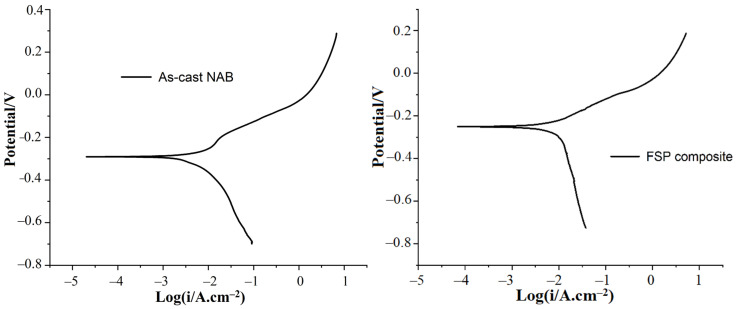
Potentiodynamic polarization curves for as-cast NAB and FS-processed composite in 3.5 wt.% NaCl solutions.

The potentiodynamic polarization test was performed to study the corrosion-resistance properties of as-cast NAB, and FSPed composite. The polarization curves were analysed to study the corrosion behaviour of the as-cast NAB and processed composite, as shown in Figure 17. The values of corrosion potential (E_corr_), polarization resistance (R_p_), Tafel slopes (b_a_,b_c_), and corrosion current density (I_corr_) are mentioned in Table 3. The table shows that the polarization resistance of the FSP composite is higher than that of the as-cast NAB. A higher value of R_p_ shows that the specimen has a higher value of corrosion resistance [61,62]. Additionally, in the case of the FSPed composite, the value of corrosion potential is higher and the corrosion current density is lower, indicating better electrochemical corrosion resistance. 

To further understand the influence of corrosion in an as-cast NAB alloy and an FS-processed composite, electrochemical impedance spectroscopy (EIS) tests were performed.

**Figure 18:**
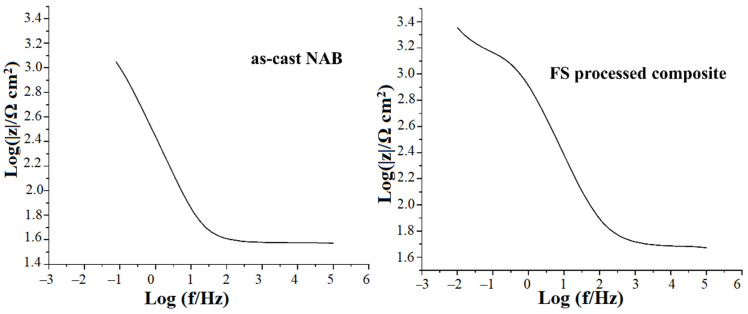
Bode plot for as-cast NAB alloy and FS-processed composite in 3.5 wt.% NaCl solutions.

The Bode plot of an as-cast NAB alloy and an FS-treated composite are shown in Figure 18. In the case of FS-processed composites, the total impedance modular |Z| increased, indicating the formation of a protective oxide film on the composite surface [63,64].

**Figure 19:**
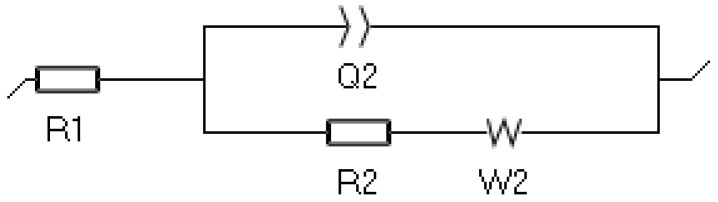
Equivalent circuit model used to fit the impedance data.

**Figure 20:**
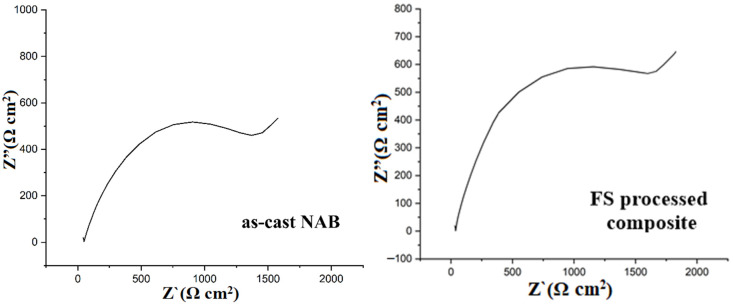
Nyquist plot for the as-cast NAB alloy and the FS-processed composite in 3.5 wt.% NaCl solution.

As illustrated in Figure 19, an equivalent circuit model built using EC lab was proposed to simulate the corrosion process. R_1_ denotes the solution resistance, R_2_ is the charge transfer resistance, Q_2_ is the constant phase element, and W is the Warburg element. Table 4 shows the fitted electrochemical parameters. The electrochemical impedance of the FS-treated composite was significantly higher than that of the as-cast NAB, as shown by Nyquist plots in Figure 20. It was believed that the coating that formed on the FS-treated composite grew quickly and was more protective. Charge transfer resistance and a constant phase element with the Warburg feature are present in all plots. The presence of a Warburg straight line indicates metal-to-ion dissolution processes, demonstrating that the corrosion of NAB alloys occurred at a predictable rate [65,66]. The diameter of the semicircle grows with the FSPed composite, suggesting that the corrosion resistance of the protective oxide film improved when compared with the as-cast NAB. 

After performing the FSP, the solution resistance value increased significantly, indicating that a more protective oxide coating formed and that, with the addition of chromium, the pore density decreased [67,68]. With the FSPed composite, the value of R_2_ increased, implying improved corrosion resistance and a reduction in redox processes. In comparison with the as-cast NAB alloy, the FSPed specimen has the highest R_1_, R_2_, and Q_2_ values, indicating that it had the highest corrosion resistance.

## 4. Conclusions

1. The normalized intensity ratio (NIR) of phases for Cu, NiAl/Fe_3_Al, and CrO_2_ were obtained as 67.63%, 21.03%, and 11.33%, respectively, from an XRD analysis of the FSPed composite.
2. The chromium-reinforced NAB composite showed a lower corrosion rate in the erosive–corrosive condition than the as-cast NAB alloy due to grain refinement in the microstructure and annihilation of the casting porosities. The weight losses in both the as-cast NAB and FSPed composite increased as the impact angle was reduced from 90° to 30°. The shear stress was predominantly at low values of the impact angle, resulting in the removal of oxide film from the surface.
3. The FSP sample had a substantially lowered static immersion corrosion rate than the as-cast NAB sample owing to grain size refinement, a reduction in the casting porosities, the formation of a Cr oxide film, and the alleviation of κ phase segregation. The corrosion rate of the as-cast NAB and FSP composite specimens decreased with an increase in the corrosion period before reaching a steady level.
4. The FSPed specimen had the highest E_corr_ and the lowest I_corr_, exhibiting the best corrosion resistance, according to the electrochemical results. With the addition of chromium using the FSP to as-cast NAB, the EIS results improved, indicating the formation of a modified oxide film with decreased pore density. The electrochemical impedance of the FSPed composite was much higher than that of the as-cast NAB, as the coating generated on the composite expanded quickly and was more protective.

## Figures and Tables

**Table 1:** Relative phase intensities of the FSPed composite.

S. No.	Phase	I_1_	I_2_	I_3_	I_back_	NIR (%)
1	Cu	37.96			3.34	67.63
2	NiAl/Fe_3_Al		14.11		3.34	21.03
3	CrO_2_			9.14	3.34	11.33

**Table 2:** Effect of impact angles on weight loss in the as-cast NAB and the FSPed composite.

Impact Angle (Degree)	Weight Loss (Mg)	
	As-Cast NAB	FSPed Composite
30	9.417	6.633
60	8.368	5.306
90	7.74	4.286

**Table 3:** Potentiodynamic polarization parameters of the as-cast NAB and the prepared composite.

Specimen	E_corr_ (V)	R_p_ (Ω·cm^2^)	b_a_ (mV·dec^−1^)	b_c_ (mV·dec^−1^)	I_corr_ (µA/cm^2^)
As-cast NAB	−0.2800	2462	126.9	247.9	14.81
FSP-prepared composite	−0.2405	5095	94.7	273.4	5.994

**Table 4:** Fitted electrochemical parameters.

Specimen	R_1_ (Ω)	R_2_ (Ω)	Q_2_ (F. s ^(a−1)^)	W (Ω cm^2^)
As-cast NAB	45.5	695	0.1534 × 10^−3^	126.5
FSP composite	46.92	1425	0.2323 × 10^−3^	189.6

## Data Availability

All data in this work is available in the form of table and figures and are available on request by contact with the corresponding author.
